# The effects of orally ingested Delta-9-Tetrahydrocannabinol on drivers’ hazard perception and risk-taking behaviours: A within-subjects study of medicinal cannabis users

**DOI:** 10.1007/s00213-025-06869-w

**Published:** 2025-07-31

**Authors:** Taren Mieran, Andrew Hill, Mark S. Horswill, Mathew J. Summers, Kayla B. Stefanidis

**Affiliations:** 1 MAIC/UniSC Road Safety Research Collaboration, University of the Sunshine Coast, 90 Sippy Downs Dr, Sippy Downs, Queensland, 4556 Australia; 2 Discipline of Psychology, School of Health, University of the Sunshine Coast, 90 Sippy Downs Dr, Sippy Downs, Queensland, 4556 Australia; 3 Minerals Industry Safety and Health Centre, Sustainable Minerals Institute, The University of Queensland, St Lucia, Brisbane, QLD 4072 Australia; 4 School of Psychology, The University of Queensland, St Lucia, Brisbane, QLD 4072 Australia

**Keywords:** Cannabis, Delta-9-tetrahydrocannabinol, THC, Road safety, Hazard perception skill

## Abstract

Medicinal cannabis use is increasing worldwide, yet its impacts on driving safety in frequent users are not clearly understood. A more comprehensive understanding of the effects of THC on driving behaviour in frequent users is needed to guide drug driving policy and evidence-based advice for medicinal cannabis consumers. This study investigated the acute effects of orally ingested THC oil on medicinal cannabis users’: (a) hazard perception skill performance; (b) driving-related risk-taking behaviours (speeding propensity, following distance, gap acceptance); (c) self-perceived hazard perception skill performance; and (d) self-perceptions of driving skills and safety. A within-subjects design was used to compare scores on validated video-based measures of hazard perception skill and risk-taking behaviours, along with self-report measures, between baseline (no THC) and post-consumption. Although participants’ (*N* = 41) actual hazard perception skill performance did not significantly decline from baseline to post-consumption, their perceived performance did (with no significant correlation between the two in either condition). In the other video-based measures, participants selected significantly slower speeds and longer following distances post-consumption (but gap acceptance behaviour was unchanged). There was no significant change in self-perceptions of driving skills and safety after correction for multiple tests. While there was no evidence that oral ingestion of THC oils by medicinal cannabis users impacted hazard perception skill performance, they were unable to accurately self-assess their performance, regardless of whether they had consumed THC. Further, medicinal cannabis patients engage in compensatory strategies, specifically by reducing their speed and increasing their following distance following the consumption of THC.

Medicinal cannabis use is increasing worldwide. For example, in Australia (where a medical model of cannabis regulation was introduced in 2016), over 700,000 applications for the use of medicinal cannabis have been approved by the Therapeutic Goods Administration as of May 2025 (Therapeutic Goods Administration 2025). These cannabis products are primarily available in a dried plant or oral form (e.g., oils, sprays, capsules) and vary in their proportions of the two primary chemical constituents of cannabis, cannabinol (CBD) and Δ9-tetrahydrocannabinol (THC; Arnold et al. 2020). While CBD is non-intoxicating, THC remains a specific compound of interest due to its psychoactive and potentially impairing properties (McCartney et al. 2022a). Despite the legalisation of medical cannabis in Australia, the country (with the exception of the state of Tasmania) has maintained a ‘zero-tolerance’ approach towards driving and testing positive to THC. However, this approach has prompted some concerns, as it has been suggested that medicinal cannabis patients can receive an infringement despite not being impaired by the substance (Love et al. 2022; Perkins et al. 2021). It is also apparent that current knowledge of the effects of THC on safety-critical driving skills and behaviours remains limited, with most research focusing on occasional cannabis users or inhaled routes of administration. Developing a comprehensive understanding of these effects will be necessary to determine the risk that THC consumption poses to road safety and has the potential to inform future drug driving policy and evidence-based advice for medicinal cannabis consumers.

Numerous studies have investigated the acute effects of THC on driving performance measures, including the capacity to maintain a consistent speed or lane position (Arkell et al. 2020b; Brooks-Russell et al. 2021; Hartman et al. 2015; Marcotte et al. 2022; Ramaekers et al. 2000). However, a review of the literature indicates that the overall effects of THC remain inconclusive, with many studies maintaining focus on inhaled routes of administration. Whilst orally ingested THC oils are a commonly prescribed solution for the treatment of conditions such as chronic pain, anxiety, and insomnia (Arnold et al. 2020; MacPhail et al. 2022), few studies have investigated the effects of orally ingested forms of THC on driving performance. Importantly, inhaled and oral methods of administration can substantially differ in onset, duration and possibly the extent of neurocognitive effects (Spindle et al. 2021). For example, whilst inhaled THC produces almost immediate physiological effects (Ramaekers et al. 2021), the peak effects of orally ingested THC do not emerge until 60–90 min post-ingestion (Curran et al. 2002; Schlienz et al. 2020; Spindle et al. 2021). Furthermore, the effects have been shown to persist for a longer duration than with inhaled methods (Curran et al. 2002; Schlienz et al. 2020; Spindle et al. 2021).

Notably, medicinal cannabis patients represent an important yet understudied population that differ in factors that may moderate acute THC-induced impairment (Ramaekers et al. 2021). First, it is possible that the alleviation of otherwise impairing symptoms (e.g., chronic pain) may mitigate the detrimental effects of THC on neurocognition and driving-related skills in this population (Ramaekers et al. 2021). However, while certain clinical populations have demonstrated improved cognitive functioning following acute THC administration (Gruber et al. 2018; Olla et al. 2021), this effect has not yet been examined using objective and validated measures of driving skills or behaviours. Second, medicinal cannabis patients have also likely developed a certain degree of tolerance to THC through the daily use of this medication. Indeed, lower consumed doses and extensive cannabis usage patterns may mitigate the effects of THC on driving performance (Bosker et al. 2012) and influence the use of compensatory driving behaviours to reduce potential safety risks, such as decreasing overall driving speed (Brooks-Russell et al. 2021; Hartman et al. 2015; Lenné et al. 2010). Despite this, there remains a paucity of research investigating the acute effects of THC on key driving-related skills and behaviours in medicinal cannabis patients. Such research is urgently needed to better understand how the effects of THC may differ in this clinical population.

In addition to objective measures of driving skills and behaviours, it is also important to consider how medicinal cannabis patients perceive their own impairment, as overestimations of driving ability are a potential risk factor for driving under the influence of cannabis (DUIC; Borodovsky et al. 2020; McDonald et al. 2021). Recent survey data reveals that medicinal cannabis patients are at a high risk of DUIC, with approximately 35% of medicinal cannabis patients driving within 3 h of consumption (Arkell et al. 2020a; Wickens et al. 2022). Prior research suggests that the most reliable predictor of this behaviour is the individual’s perception of the safety of the behaviour (Borodovsky et al. 2020; Jones et al. 2007; Malhotra et al. 2017; McDonald et al. 2021). Cannabis users who inaccurately overestimate their driving ability while in a state of THC-induced neurocognitive intoxication are therefore more likely to DUIC. However, most medicinal cannabis patients believe that they can accurately judge their own level of impairment (Arkell et al. 2020a). Whilst preliminary evidence indicates that cannabis users may be limited in their ability to accurately appraise their own driving capacity (Arkell et al. 2019, 2020b; Marcotte et al. 2022), no studies have yet directly examined the relationship between self-rated and objective measures of driving performance following THC consumption. According to Sundström (2008), establishing the accuracy of performance appraisals requires a direct comparison between objective skill and subjective ratings. Examining this association using a validated measure of a key safety-critical driving skill may therefore help to establish the extent to which cannabis users can accurately appraise their own driving performance while acutely influenced by THC. Drivers’ hazard perception (i.e., the ability to anticipate dangerous situations on the road ahead) is an appropriate focus for such an investigation because it is one of the few driving skills that has consistently been found to predict crash risk (Horswill 2016; Horswill and Hill 2021).

To further understand how THC affects driving capacity, the present study addressed five research questions about the acute effects of orally ingested THC oil in a sample of medicinal cannabis users:

- **RQ1.** Does THC oil ingestion affect drivers’ objective hazard perception skill performance?
- **RQ2.** Does THC oil ingestion affect driving-related risk-taking behaviours?
- **RQ3.** Does THC oil ingestion affect drivers’ subjective perceptions of their own hazard perception skill performance?
- **RQ4.** Do THC oil users’ subjective perceptions of their own hazard perception skill performance correlate with objective performance?
- **RQ5.** Does THC oil ingestion affect drivers’ perceptions of their own on-road driving skills and safety?

To address these questions, the study used validated video-based tests of hazard perception skill (RQ1) and three key risk-taking behaviours– speeding propensity, following distance, and gap acceptance (RQ2). Self-report measures were used to assess drivers’ perceptions of their hazard perception skill performance (RQ3) and on-road driving skills and safety (RQ5). Scores on each of the video-based measures have been shown to be associated with crash risk, risky real-world driving behaviours, or a key correlate thereof (Horswill et al. 2010, 2015, 2020, 2022). Hence, the present study can potentially provide an important contribution to developing a more comprehensive understanding of the effects of THC on driving behaviour, as well as inform the future development of impairment-based detection methods for drug driving involving THC.

## Method

### Participants

Forty-three adult medicinal cannabis patients were recruited from the Sunshine Coast in Queensland, Australia, through Facebook advertising and medicinal cannabis clinics. The study inclusion criteria required that participants were aged 18 years or older, held a current Queensland driver’s licence, drove at least once per week, and held a valid prescription for orally ingested cannabis oil containing THC. Potential participants were deemed ineligible if they reported uncorrected visual or hearing impairments, diagnosed neurological impairment or disease (e.g., traumatic brain injury, mild cognitive impairment, and dementia), diagnosis of a major psychiatric illness (including schizophrenia, delusional disorder or panic disorder), epilepsy, or current pregnancy. Two participants withdrew from the study prior to completing their second session, leaving a final sample of 41 for data analysis. All participants indicated consent online when volunteering and provided written consent on the day of testing. This research was approved by the University of the Sunshine Coast Human Research Ethics Committee (approval A211677) and complied with the National Health and Medical Research Council (NHMRC) of Australia ethical guidelines. Clinical trial registration was not deemed necessary for this study given that participants were consuming their own medication at doses consistent with their treatment plan.

### Materials

#### Hazard perception test

**Fig. 1:**
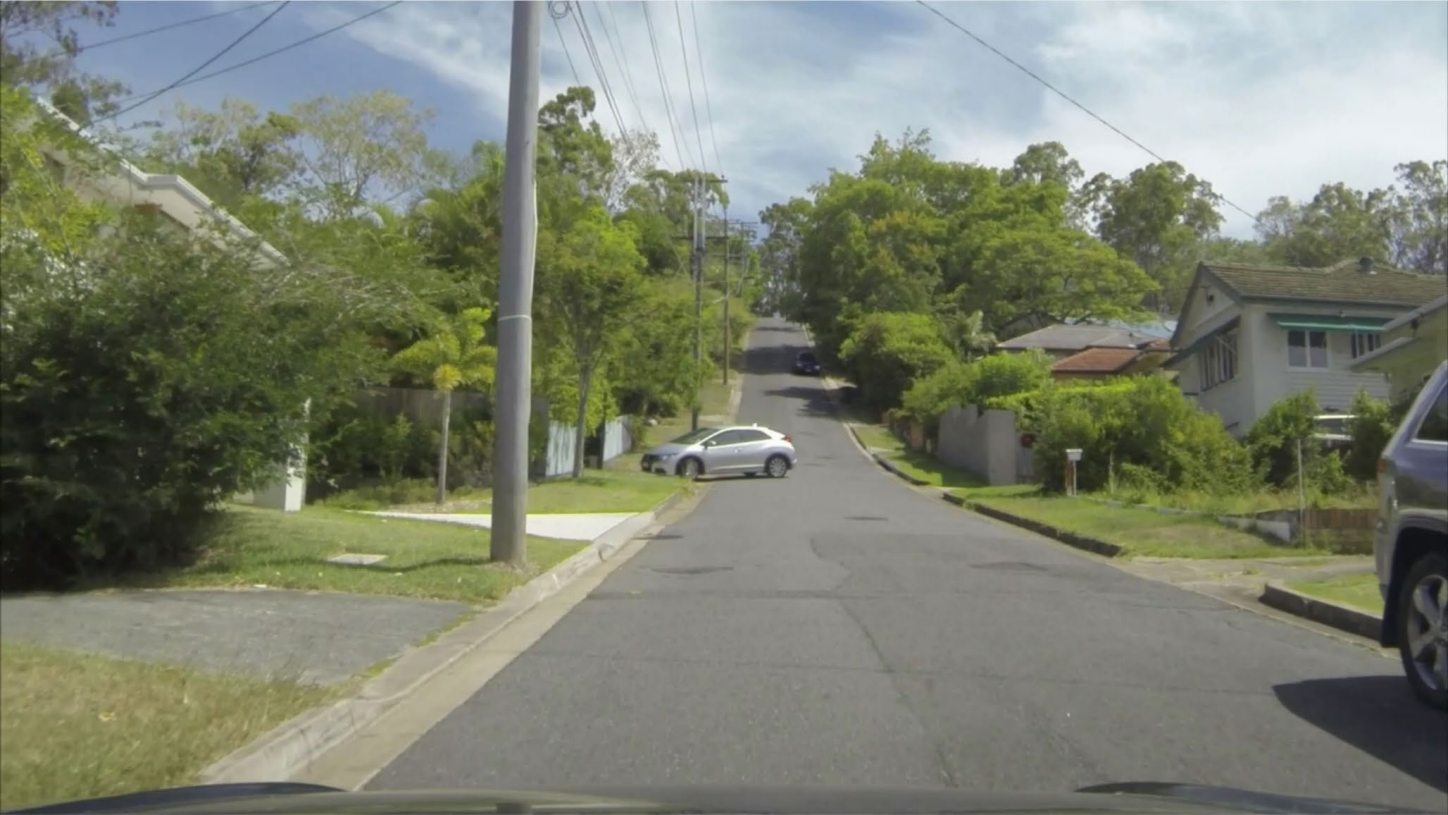
Still image from a video clip used in the Hazard Perception Test showing the road ahead from the driver’s perspective with a traffic conflict underway

The Hazard Perception Test (HPT) is a computerised assessment that measures a driver’s ability to anticipate traffic conflicts (i.e., hazard perception skill; Hill et al. 2019). The test items comprise a series of video clips of genuine traffic footage (see Fig. 1), filmed from the driver’s perspective, which contain traffic conflict scenarios (i.e., situations in which it is necessary to slow down or change course to prevent a crash). The participant’s task is to predict each traffic conflict as early as possible, and to use a computer mouse to indicate their prediction immediately by clicking on the other road user (or users) involved in the traffic conflict. The outcome measure (i.e., the overall test score) is the participant’s average response time in seconds (after item score standardisation) to identify the traffic conflicts contained in the clips. Alternate forms of the test, containing different clips (30 per test), were administered to each participant for the baseline and post-consumption conditions to prevent practice effects. Administration was counterbalanced such that approximately half of the sample completed each version at each timepoint.

Half of the 60 clips used in the present study were from the 30-item hazard perception test developed by Hill et al. (2019). Overall scores on this test were found to predict the frequency of heavy braking during everyday driving. These 30 clips were drawn from an initial pool of 57 clips, which were found, collectively, to significantly differentiate higher-risk (novice) and lower-risk (experienced) driver groups (as were overall scores on the final 30-item test). An additional 8 clips from this pool were also used in the present study, and the remaining 22 clips were taken from a pool of new items created for an unpublished study (Kieseker 2022). These 30 items were selected based on correlations with overall scores on Hill et al.’s (2019) 30-item test in Kieseker’s (2022) sample. In the present study, each test included 15 of these clips and 15 from Hill et al.’s (2019) final test. All 60 clips used in the present study were conceptually similar to clips created by the same researchers using the same methodology for other hazard perception tests for which overall scores have been found to distinguish novice from experienced drivers and (where evaluated) to predict crash involvement (e.g., Horswill et al. 2010; Horswill et al. 2015; Horswill et al. 2008; Manley et al. 2020; Wetton et al. 2011). For a detailed explanation of this approach to hazard perception test development methodology, see Wetton et al. (2011).

Note that direct comparisons between the hazard perception test scores obtained in the present study and those of participant groups from previous datasets would be potentially misleading due to factors such as variations in sample characteristics and study protocols. In particular, the wide variation in age and driving experience of participants in the present study renders any such comparisons uninterpretable. Nevertheless, these issues are irrelevant to the within-participant comparisons made in the present study.

#### Following distance test

**Fig. 2:**
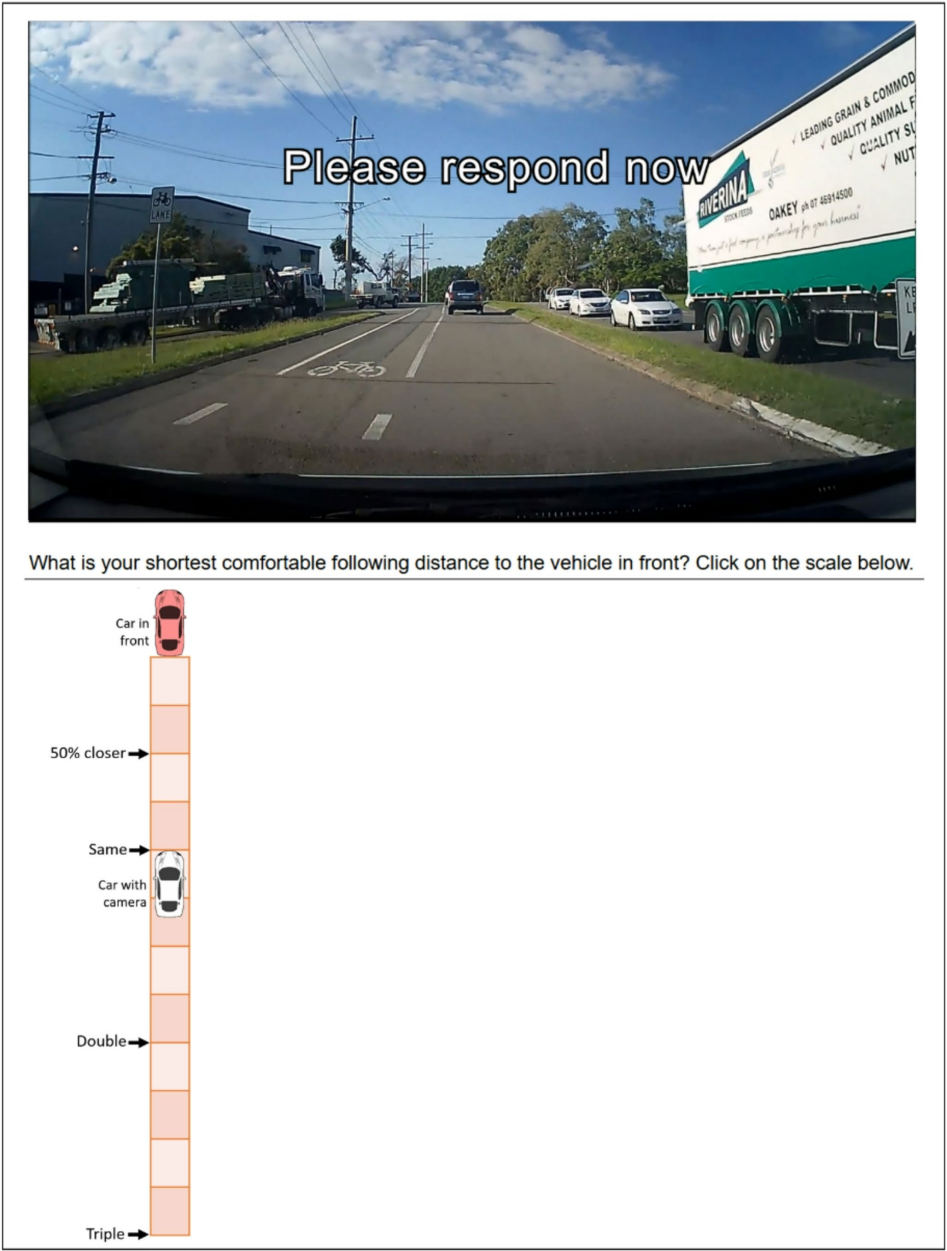
Screenshot from the Following Distance Test showing the final frame of a video clip and the response scale

The Following Distance Test is a validated proxy measure of a driver’s following distance behaviour (Horswill et al. 2020). The test presents the participant with 20 video clips of genuine traffic footage, filmed from the point of view of a driver. The clips depict various situations in which the driver is following other vehicles at a range of distances (Horswill et al. 2020). The participant is required to indicate the extent to which their own “minimum comfortable following distance” differs from the following distance depicted in each scenario. Responses are recorded on a vertical visual analogue scale (VAS) that allows the participant to place a mark at any point between the rear of the leading vehicle and a point that represents triple the depicted following distance (with anchor points labelled ‘50% closer’, ‘same’, ‘double’, and ‘triple’). See Fig. 2 for an example item. To generate the outcome measure for each participant, their responses are converted to following distances in seconds and averaged across all scenarios.

#### Video speed test

**Fig. 3:**
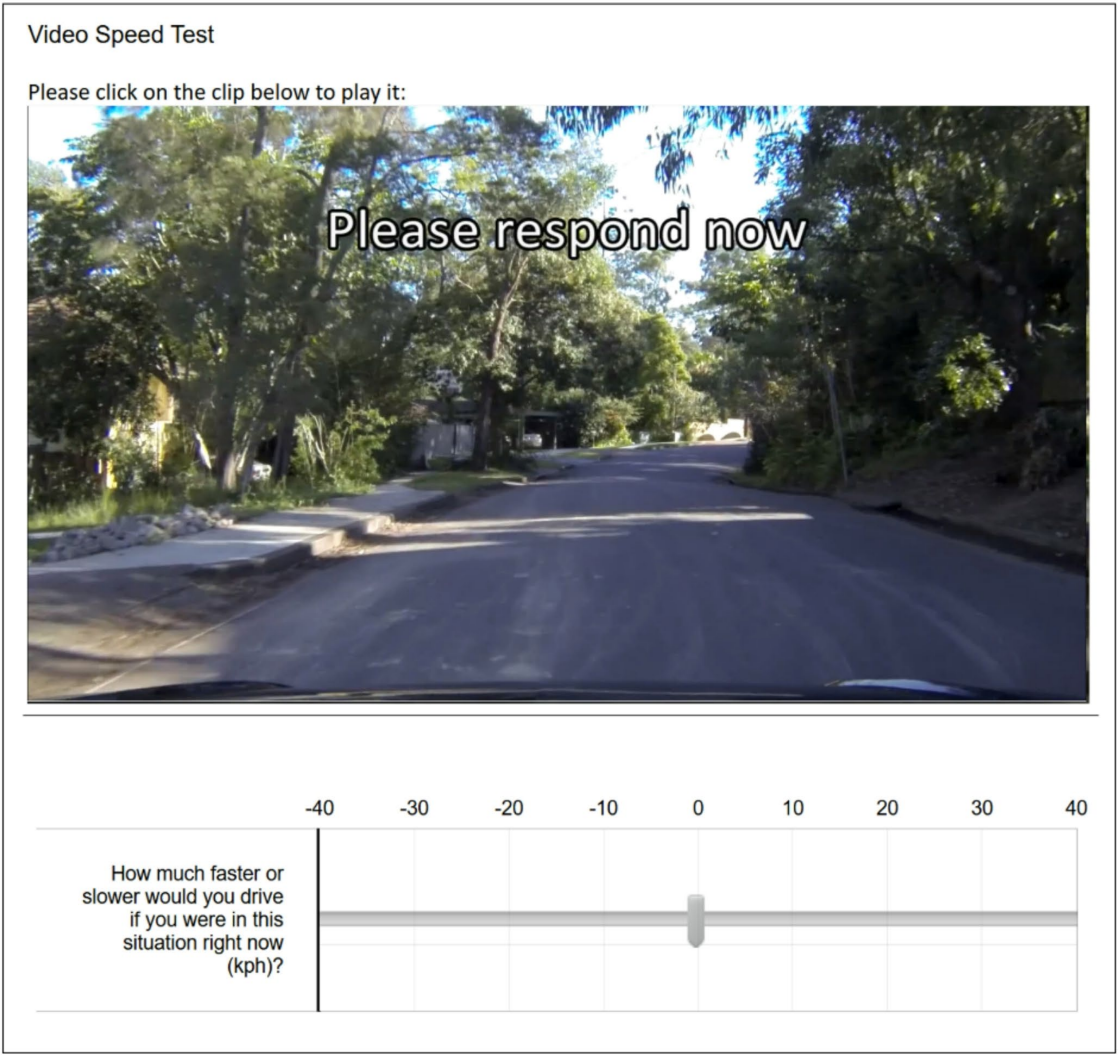
Screenshot from the Video Speed Test showing the final frame of a video clip and the response scale

The Video Speed Test provides a measure of speeding propensity and can be used as a reliable proxy for real-world speeding behaviour (Horswill et al. 2022). It presents 16 video clips shot from the perspective of the driver of a forward-moving vehicle (Horswill et al. 2022). No other vehicles or obstacles that may prevent the vehicle from traveling faster are included in any of the scenarios. Participants use a horizontal VAS to indicate the extent to which they would drive faster or slower than the camera vehicle in each scenario in km/h (see Fig. 3). Participants are also provided with an initial practice clip before beginning the test. No quantitative information on the vehicle’s true speed (e.g., speedometer) is given to participants. The outcome variable is the average of the participant’s responses across all scenarios.

#### Gap acceptance test

**Fig. 4:**
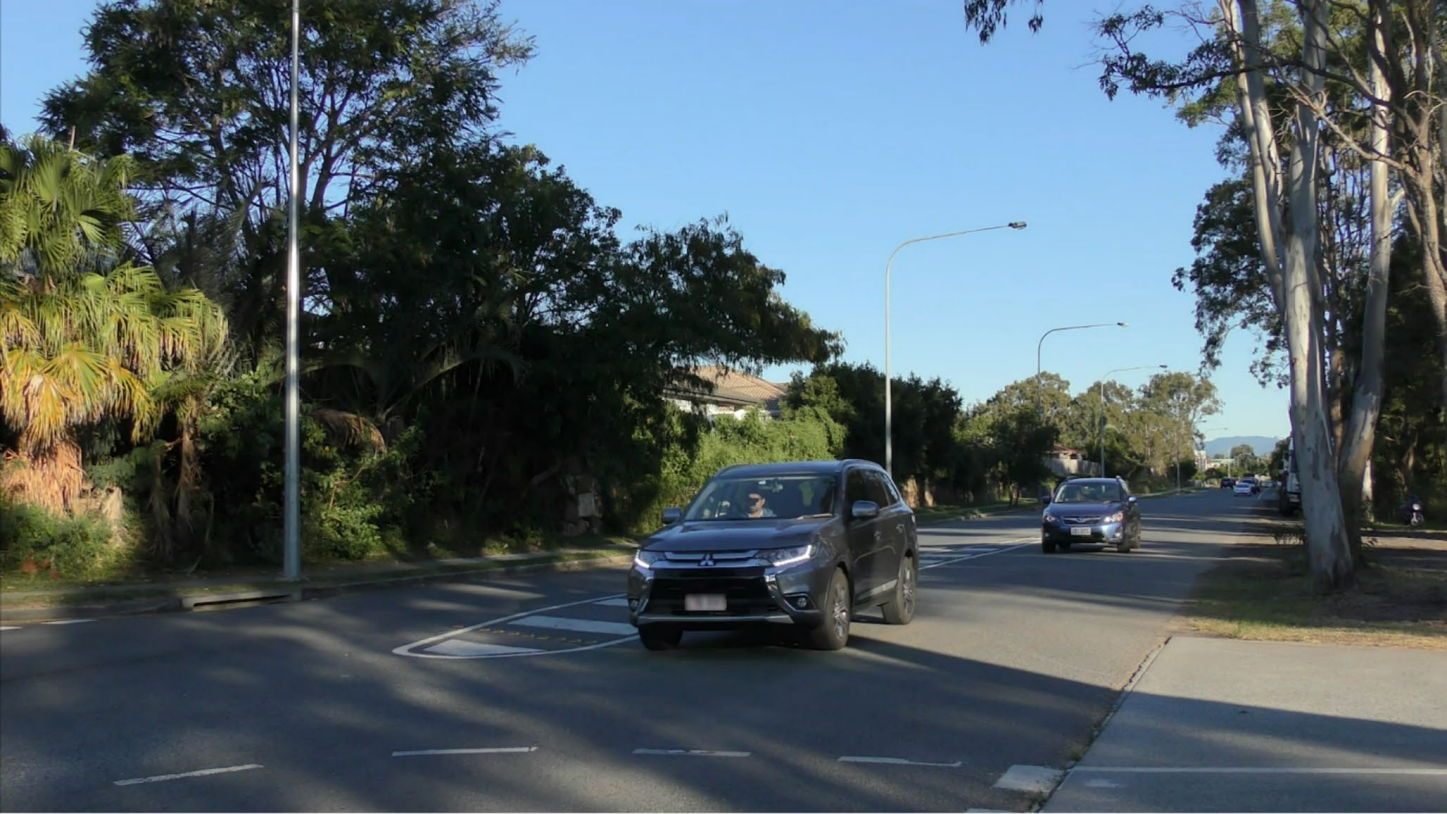
Still image from a video clip used in the Gap Acceptance Test showing a stream of oncoming traffic from the perspective of a driver waiting to turn left (i.e., looking through the side window)

The Gap Acceptance Test is a validated proxy measure of a driver’s gap acceptance behaviour (Horswill et al. 2020). The test presents 23 video clips that display a stream of oncoming traffic from the perspective of the driver of a small vehicle waiting to turn left into a major road from a minor road (Horswill et al. 2020). See Fig. 4 for an example clip. During each clip, the participant’s task is to click with the computer mouse when presented with a gap that they would be willing to pull out into. Each clip ended with a long gap (at least 6.83s, a length that nearly all drivers would accept), which is preceded by a sequence of shorter gaps of varying length. When the participant clicks to indicate that they would pull out, the clip playback stops, and the next clip starts immediately. If the participant reaches the end of a clip without responding, the next clip starts automatically. The outcome measure is the mean time elapsed (in seconds) between the beginning of each clip and the participant’s response (or the end of the clip, if the participant did not respond).

#### Visual analog scale for self-ratings of hazard perception skill performance

After completing each HPT, participants rated their own performance on the test from 0 to 100 on a horizontal VAS (adapted from Horswill et al. 2013). Specifically, these ratings were made in response to the question, “This question is about the Traffic Conflict Prediction Assessment that you just completed. Please give your best guess. How early did you predict the traffic conflicts compared with other Sunshine Coast drivers (0 = worst driver, 50 = typical driver, 100 = best driver)?”

#### Visual analog scales for self-ratings of on-road driving skills and safety

In each condition (baseline and post-consumption) participants completed a state-based measure of their own driving skills and safety (also adapted from Horswill et al. 2013). The measure comprised four horizontal VAS items. Participants were asked, “If you were driving right now, how would you compare to other Sunshine Coast drivers (0 = worst, 50 = typical, 100 = best) for each of the following?” The items were *predicting traffic conflicts*, *overall driving skill*, *overall driving safety*, and *crash risk.*

#### Subjective drug effects

A 17-item Drug Effects Questionnaire (DEQ; adapted from Spindle et al. 2021) was administered to characterise subjective drug effects in the post-consumption condition. The questionnaire included items that represent both positive (e.g., “like drug effect”) and negative subjective effects (e.g., “dislike drug effects”), mood states (e.g., “anxiousness”, “paranoia”), and perceived level of impairment (e.g., “difficulty concentrating”, “difficulty with routine tasks”). Responses are marked on each item using a VAS ranging from 0 (“not at all”) to 100 (“extremely”).

### Design and procedure

**Fig. 5:**
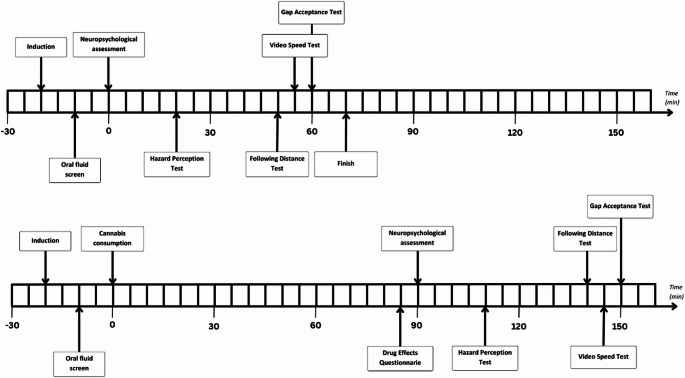
Timeline of baseline (top) and post-consumption (bottom) session procedures. Note each block represents a 5-minute segment of time.

Each participant took part in two testing sessions (*baseline* and *post-consumption*), which were scheduled approximately one week apart. The order of the conditions was counterbalanced, and participants were asked to maintain an 11.5-hour abstinence from any cannabis usage prior to each session to minimise the likelihood of residual neurocognitive effects from prior THC consumption (McCartney et al. 2021). Testing sessions commenced at either 8:30am or 1:00pm depending on the participants’ medication schedule, and participants were required to complete both of their sessions at the same time of day. Figure 5 provides a high-level timeline of the study procedures for the baseline and post-consumption sessions.

Prior to their first appointment, participants completed an online questionnaire to gather information on demographics, medical history, body composition, pharmaceutical medications, and drug use history. Upon their arrival for each session, a researcher visually inspected the participant’s prescription to ensure it was valid and current, and recorded the prescribed dose. Oral fluid was also screened (Dräger DrugCheck 3000) to identify the recent use of psychoactive substances including THC, amphetamines, methamphetamines, opiates, benzodiazepines, and cocaine.

During the baseline session, participants completed all assessments while refraining from any cannabis usage. During the post-consumption session, participants completed all assessments following the oral (on top of tongue) or sublingual (under tongue) consumption of a single dosage of their prescribed THC oil product. A researcher visually examined the dropper before consumption to ensure it did not exceed their prescribed dose. A 90-minute rest period was required between THC consumption and the assessment, as determined by McCartney et al.’s (2021) meta-regression analysis of predicted peak effects for orally ingested THC among regular cannabis users. Before beginning the video-based driving assessments, participants also completed a comprehensive neuropsychological battery that lasted approximately 20 min (the results of which are to be reported elsewhere). All driving assessments were completed on a laboratory PC with wireless headphones. If the participant normally wore glasses while driving, they were instructed to wear them during the assessments. Complimentary taxi transport was organised to transfer participants to and from the laboratory.

### Statistical analysis

All statistical analyses were conducted using SPSS Version 28.0 (IBM Corp 2022). Descriptive statistics were computed for participant characteristics, saliva test results, cannabis usage history, cannabis consumption, and subjective drug effects. Variables related to typical THC consumption (e.g., THC consumed per day via oil) were calculated using self-reported data on product THC concentrations, typical single session doses, and uses per day. To examine changes in driving task performance and self-ratings from baseline to post-consumption, paired samples *t*-tests were conducted. Shapiro-Wilk tests revealed that most outcome measures were normally distributed. However, several distributions breached the assumption of normality, and bootstrapped *t*-tests (bias corrected and accelerated 95% confidence intervals) were therefore conducted for all analyses (Field 2018). For research questions with multiple associated significance tests, the Bonferroni-Holm correction was applied to control the familywise error rate (Holm 1979). The magnitude of differences between conditions was quantified using Cohen’s *d*, with 0.2 representing a small effect, 0.5 a moderate effect, and 0.8 indicating a large effect (Cohen 1988). Due to the small sample size and substantial overlap between treating conditions (e.g., chronic pain patients also treating mental health and sleep), differences between medical condition types were not explored. A 2 × 2 mixed *ANOVA* was conducted to examine hazard perception performance as a function of both testing condition (within-subjects factor; baseline, post-consumption) and THC dose (between-subjects factor; lower and higher). Participants were placed into lower and higher THC dose groups based on a median split of consumed THC dose (mg per kilogram of bodyweight). Finally, Pearson correlations were conducted to examine the association between self-rated and objective hazard perception performance at both baseline and post-consumption. Pairwise deletion was used to remove one participant from any analyses involving the Following Distance Test, Video Speed Test, and self-ratings as their post-consumption data was missing due to technical issues.

## Results

### Participant characteristics

Information pertaining to participant demographics, cannabis usage histories, and current alcohol use is presented in Table 1. The final sample comprised 41 participants, with an age range of 21–67 years. Two additional participants were included in the study but withdrew prior to completing their second session. All participants held an open/unrestricted driver’s licence. Sample characteristics related to sex, age, treating condition, and product type (e.g., oil, flower) are generally consistent with available data on Australian medicinal cannabis Special Access Scheme Category B approvals (Therapeutic Goods Administration 2024).

**Table 1:** Characteristics of participants

Variable	*n* or M (SD)	% or range
Sex		
Male	23	56.1%
Female	18	43.9%
Age	46 (13)	21–67
BMI	27.85 (5.47)	18.6–44.1
Time since passing on-road driving test (years)	27.9 (13.3)	3–48
Average kilometres driven per week	309.3 (261.0)	39.0-1200.0
Diagnosed psychiatric disorder	19	46.3%
Depression	11	26.8%
Anxiety	11	26.8%
Bipolar disorder	1	2.4%
PTSD	6	14.6%
ADHD	2	4.9%
Not specified	2	4.9%
Health conditions (current or historical)		
Respiratory condition	3	7.3%
Cancer	2	4.9%
Cardiovascular/heart disease	2	4.9%
Physical injury	12	29.3%
Type II diabetes	3	7.3%
Kidney disease	1	2.4%
Multiple sclerosis	1	2.4%
Other (e.g., low vitamin B12)	2	4.9%
Medicinal cannabis treating condition*		
Chronic pain	28	68.3%
Mental health	20	48.8%
Sleep issues	15	36.6%
Gastrointestinal	2	4.9%
Cancer symptoms	1	2.4%
Other (e.g., chronic migraines)	4	9.8%
Medications		
Antidepressants	11	26.8%
Anxiolytics	6	14.6%
Anticonvulsants	3	7.3%
Blood pressure	5	12.2%
Opiates	7	17.1%
Anti-inflammatory	9	22.0%
Gastrointestinal	5	12.2%
Other (diabetes, hormone replacement)	4	9.8%
Time prescribed THC oil (months)	10 (11)	1–41
THC oil uses per day		
1	20	48.8%
2	18	43.9%
3–4	2	4.9%
5+	1	2.4%
Prescription for other medicinal cannabis products		
THC flower	27	65.9%
CBD oils	5	12.2%
THC capsules	1	2.4%
Self-reported typical THC consumption		
Single session oil THC dose (mg)	19.57 (24.56)	0.02-100
Single session flower THC dose (mg)	67.31 (79.00)	0-250
THC consumed per day via oil (mg)	35.75 (64.13)	0.04-375
THC consumed per day via flower (mg)	144.07 (153.70)	0-500
THC consumed per day via oil and/or flower combined (mg)	179.81 (178.13)	0.12–625
Prior use of illicit cannabis	16	39.0%
Overall cannabis use time (years)	15 (16)	0–48
Past month cannabis use (days)	26 (8)	2–31
Current alcohol use		
Weekly	21	51.2%
Daily	9	22.0%

*N* 41, *ADHD* attention-deficit hyperactivity disorder, *BMI* Body Mass Index, *CBD* cannabidiol, *PTSD* post-traumatic stress disorder, *THC* Delta-9-tetrahydrocannabinol

*Note participants self-reported the condition(s) that medicinal cannabis was prescribed to treat so this information was not derived from verifiable medical records.

### Saliva tests

The proportion of positive saliva test results for each psychoactive substance at the beginning of each testing session is presented in Table 2. All participants confirmed that they had not consumed cannabis for at least 11.5 h prior to testing. One participant tested positive to both methamphetamines (post-consumption) and cocaine (baseline and post-consumption). To test whether the inclusion of this participant influenced the results, a sensitivity analysis was run with and without this participant on all *t*-tests. Removing this participant from the analyses did not change any results.

**Table 2:** Frequencies of positive saliva test results for tests administered at the beginning of each testing session

Substance type	Baseline session		Post-consumption session		Both sessions	
	*N*	%	*N*	%	*N*	%
THC	19	46.3%	20	48.8%	14	34.1%
Amphetamines	0	0%	1	2.4%	0	0%
Methamphetamines	0	0%	1	2.4%	0	0%
Opiates	2	4.9%	4	9.8%	2	4.9%
Benzodiazepines	1	2.4%	1	2.4%	1	2.4%
Cocaine	1	2.4%	1	2.4%	1	2.4%

*N* = 41. THC = Delta-9-tetrahydrocannabinol

## Cannabis consumption

During the post-consumption session, participants consumed a mean of 10.80 mg THC (*SD* = 11.95, range = 0.06–50), and a mean of 16.05 mg cannabidiol (*SD* = 54.58, range = 0-350). This equated to a mean of 0.12 mg THC per kg of bodyweight (SD = 0.12, range = 0.00-0.59). A mix of sativa dominant (*n* = 10, 24.4%), indica dominant (*n* = 7, 17.1%), and hybrid (*n* = 2, 4.9%) plant origin strains were consumed by participants. However, the strain was unclear for 22 (53.7%) of the products. Note many medicinal cannabis oils available in Australia are THC or CBD isolates in a carrier oil, and therefore are not derived from a specific strain. Thirty-eight (92.7%) participants consumed the oil product using a sublingual administration method, whereas three (7.3%) participants used an oral administration method. There were no adverse reactions to cannabis during any of the post-consumption sessions.

## Effect of THC oil ingestion on drivers’ hazard perception skill performance

**Fig. 6:**
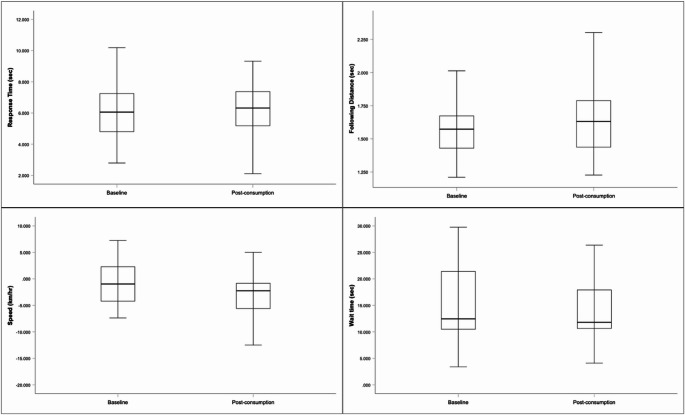
Boxplots of performance on the hazard perception test (top left), following distance test (top right), video speed test (bottom left), and gap acceptance test (bottom right) at baseline and post-consumption

Figure 6 displays boxplots for each of the video-based driving measures at baseline and post-consumption. A paired-sample *t*-test (*N* = 41) revealed no significant change in participants’ scores on the video-based hazard perception test between the baseline (*M* = 6.233 s, *SD* = 1.717) and post-consumption (*M* = 6.144 s, *SD* = 1.735) conditions, *p* =.644, 95% bias-corrected and accelerated confidence interval (BCBCI) [−0.47, 0.30], *d* = 0.07.

**Fig. 7:**
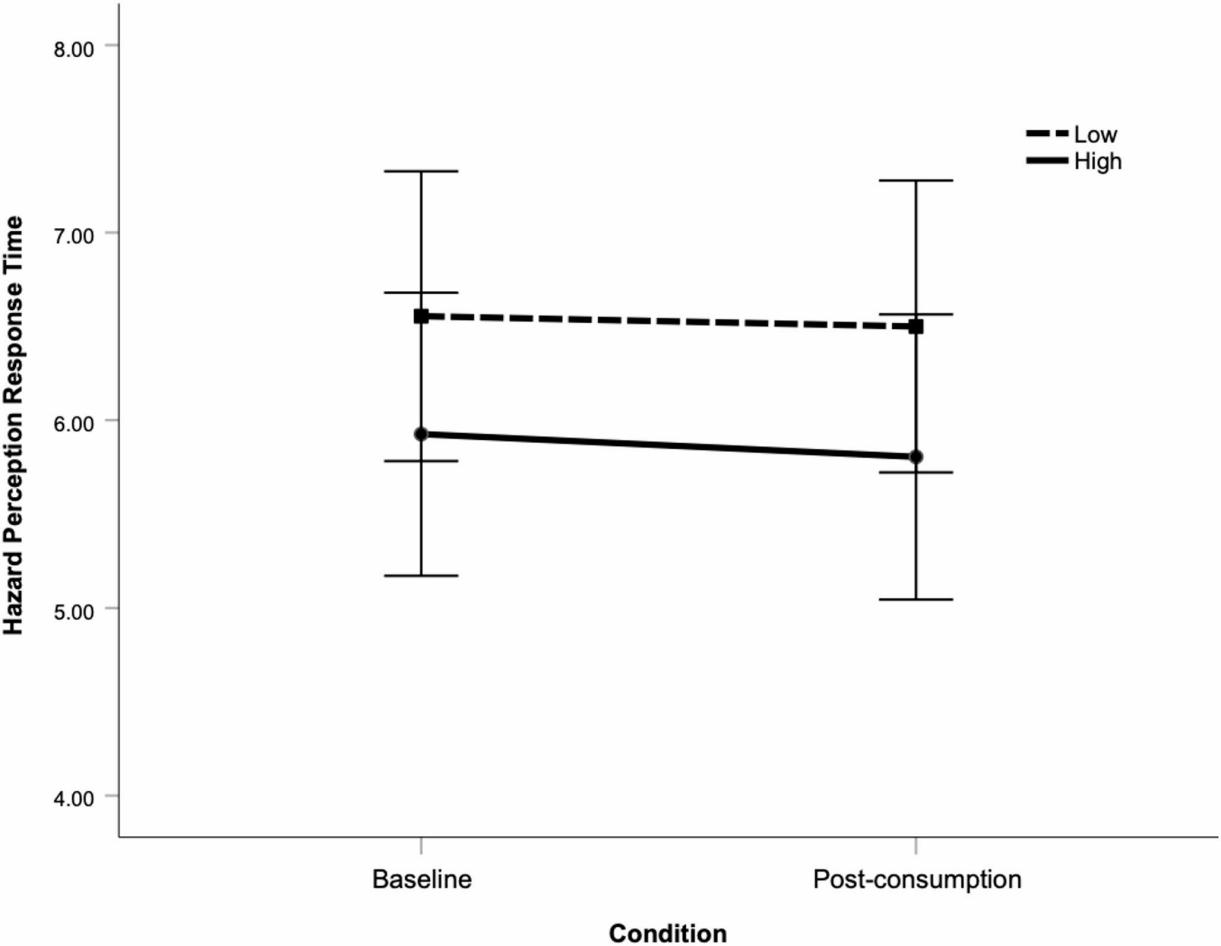
Mean hazard perception response times (sec) as a function of condition and THC dose. Note. Low and high THC dose calculated via median split of consumed THC mg/kilogram of body weight.

A two-way mixed ANOVA was also conducted to examine whether hazard perception response time differed as a function of testing condition (baseline vs. post-consumption) or dose group (median split into higher vs. lower THC mg/kilo dose). This analysis revealed no significant main effect for condition, *p* =.667, *F*(1, 39) = 0.188, or dose, *p* =.189, *F*(1, 39) = 1.784. The interaction effect was also non-significant, *p* =.871, *F*(1, 39) = 0.027. Levene’s test confirmed homogeneity of variance. Figure 7 displays mean hazard perception response times as a function of condition and THC dose.

## Effects of THC oil ingestion on driving-related risk-taking behaviours

Table 3 presents the means, standard deviations, and paired sample *t*-test results for changes in participants’ driving-related risk-taking behaviours from baseline to post-consumption, as measured using video-based tests. There was a significant increase in participants’ comfortable following distance, and a significant decrease in their preferred speed from baseline to post-consumption, following a Bonferroni-Holm correction. There was no significant change in gap acceptance wait time.

**Table 3:** Video-based measures of driving-related risk-taking behaviours at baseline and post-consumption

Measure	Baseline	Post-consumption	Paired sample t-test (2-tailed)		Effect size
	*M (SD)*	*M (SD)*	*p*	95% *BCBCI*	*d*
Following Distance Test (*N* = 40)	1.537 (0.188) sec	1.645 (0.325) sec	0.019*	[0.05, 0.17]	0.50
Video Speed Test (*N* = 40)	−1.058 (4.447) km/hr	−3.302 (5.106) km/hr	< 0.001*	[−3.15, −1.41]	0.73
Gap Acceptance Test (*N* = 41)	14.935 (6.837) sec	14.580 (5.780) sec	0.534	[−1.97, 1.00]	0.10

*Significant with Bonferroni-Holm correction. BCBCI = bias-corrected and accelerated confidence interval

## Effect of THC oil ingestion on participants’ self-ratings of hazard perception skill performance

A paired-sample *t*-test (*n* = 40) revealed a significant decrease in participants’ self-reported perceptions of their own hazard perception test performance from the baseline condition (*M* = 76.93, *SD* = 14.01) to the post-consumption condition (*M* = 71.45, *SD* = 15.18), *p* =.031, 95% BCBCI [−0.10.06, −1.20], *d* = 0.40.

## Effects of THC oil ingestion on participants’ self-ratings of on-road driving skills and safety

Table 4 presents the means, standard deviations, and paired sample *t*-tests results for changes in participants’ self-ratings of their on-road driving skills from baseline to post-consumption. There was no significant difference for any of the self-rating measures after applying a Bonferroni-Holm correction.

**Table 4:** Self-ratings of on-road driving skills and safety at baseline and post-consumption

Measure	Baseline	Post-consumption	Paired sample t-test (2-tailed)		Effect size
	*M (SD)*	*M (SD)*	*p*	95% *BCBCI*	*d*
Predicting traffic conflicts	77.03 (17.36)	68.22 (22.03)	0.022	[−16.99, −1.44]	0.38
Driving skill	76.90 (18.08)	69.83 (23.11)	0.076	[−15.22, 0.41]	0.30
Driving safety	79.72 (17.95)	71.00 (23.50)	0.042	[−18.05, − 0.13]	0.34
Crash risk	81.80 (18.66)	73.68 (24.75)	0.096	[−18.61, 0.98]	0.27

*N* = 40. Self-ratings measured using Visual Analogue Scales where 0 = worst driver, 50 = average driver, and 100 = best driver

## Associations between objective and self-rated hazard perception skill performance

Pearson correlations revealed no significant association between participants’ hazard perception response times and their self-rated hazard perception performance at baseline, *r*(41) = 0.21, *p* =.190, or post-consumption, *r*(40) = 0.17, *p* =.296.

## Subjective drug effects

**Fig. 8:**
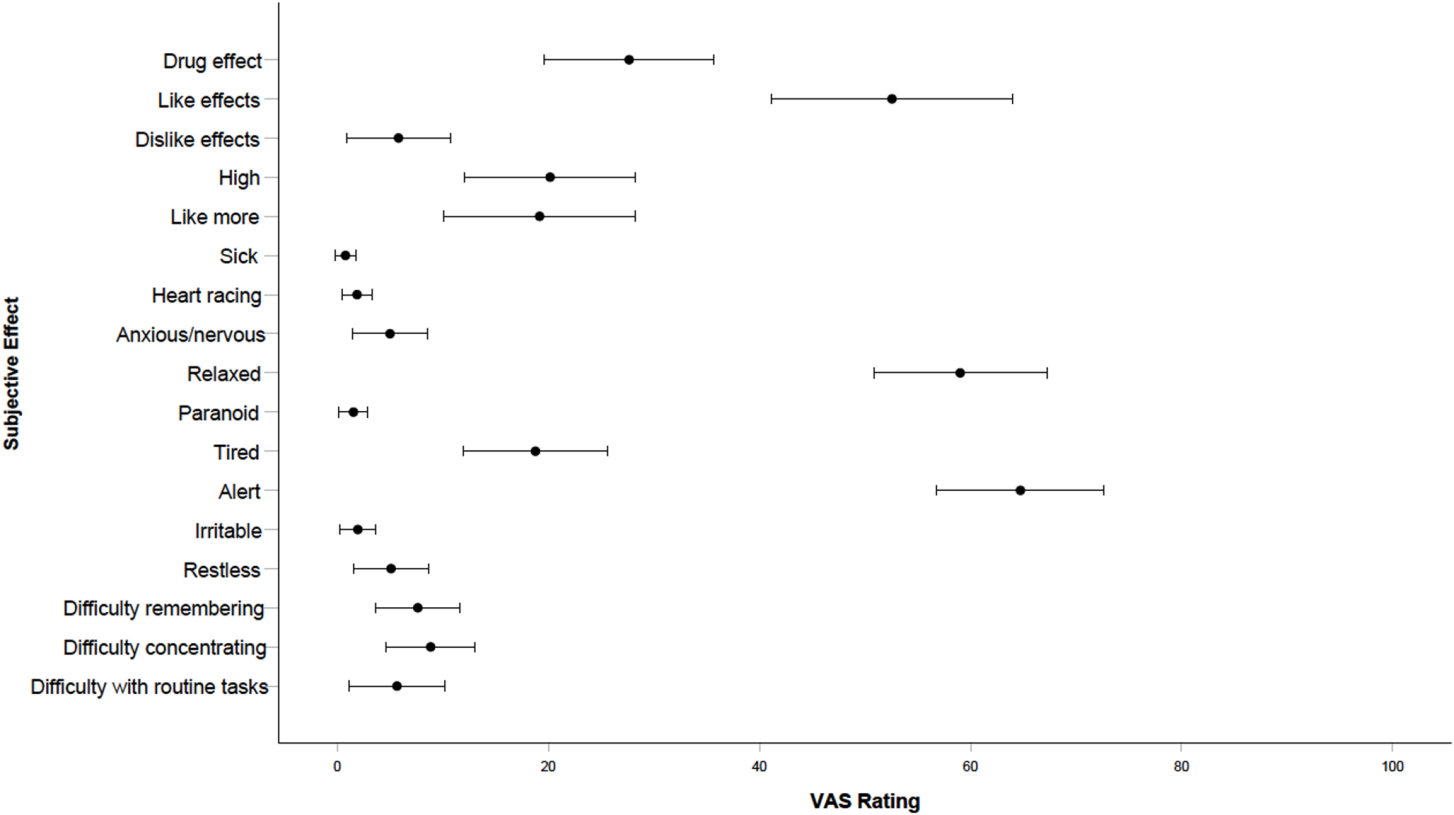
Error bars for subjective drug effects ratings at 85-minutes post-consumption

Subjective drug effects VAS ratings at 85-minutes post-consumption are visually presented in Fig. 8. Participants reported a mean “drug effect” of 27.66 (*SD* = 25.53), and a mean subjective “highness” of 20.17 (*SD* = 25.68). VAS ratings for “liking drug effects” (*M* = 52.56; *SD* = 36.18), “relaxed” (*M* = 59.02; *SD* = 25.98), and “alert” (*M* = 64.73; *SD* = 25.13) were rated the highest of all drug effects.

## Discussion

The present study investigated the acute effects of orally ingested THC oil on medicinal cannabis patients’: (a) hazard perception skill performance; (b) driving-related risk-taking behaviours (i.e., speeding propensity, following distance, and gap acceptance); (c) self-ratings of their own hazard perception skill performance (including the association between subjective self-ratings and objective performance); and (d) self-ratings of their own on-road driving skills and safety. Although participants’ actual hazard perception skill performance did not significantly decline from baseline to post-consumption, their perceived performance did. Moreover, there was no significant correlation between self-rated and objective hazard perception skill performance at either baseline or post-consumption. In the video-based measures of driving-related risk-taking behaviours, participants selected significantly slower speeds and longer following distances post-consumption of THC, although there was no significant change in their gap acceptance behaviour. There was also no significant change in self-perceptions of current on-road driving skills and safety after correction for multiple tests. Overall, while the study yielded no evidence that oral ingestion of THC oils by medicinal cannabis patients impacted hazard perception skill performance, the results suggest that these consumers are unable to accurately self-assess their hazard perception skill performance, regardless of whether they have consumed THC. This lack of insight into one’s own driving skill corresponds to findings for other driver groups (Groeger and Grande 1996; Horswill et al. 2013). Further, our findings suggest that medicinal cannabis patients engage in compensatory behaviours (specifically, by reducing their speed and increasing their following distance). Such findings provide important insights into the impact of orally ingested THC on driving behaviour and has the potential to inform future drug driving policy and evidence-based advice for medicinal cannabis consumers.

As noted above, the present study yielded no significant difference in hazard perception skill performance between baseline and post-consumption of THC oil, which may suggest that hazard perception ability is unaffected by the acute administration of orally ingested THC amongst medicinal cannabis patients. To date, only two studies have examined the effects of orally ingested THC on driving performance (Bosker et al. 2012; Manning et al. 2024). Bosker et al. (2012) found 20 mg of oral synthetic THC (dronabinol) increased on-road SDLP (standard deviation of lateral position) by 4.2 cm relative to placebo, with peak effects occurring at 90-minutes post-ingestion. However, this finding is based on a small sample of 12 occasional recreational cannabis users. Using a naturalistic design similar to the present study, Manning et al. (2024) examined the effects of prescribed medicinal cannabis oils on simulated driving performance in Australian medicinal cannabis patients. Consistent with our findings, they found no notable evidence of driving performance decrements relative to baseline.

Our finding of no significant difference in hazard perception skill performance is interesting, given that a substantial portion of variance in hazard perception performance is explained by key cognitive functions (i.e., processing speed, attention, psychomotor ability, and executive function) that have previously been shown to be affected by THC (Horswill et al. 2008; McInerney and Suhr 2016). One potential explanation is that it may be attributable to a high tolerance level amongst medicinal cannabis patients. Specifically, the present sample had an average cannabis usage history of 15 years and consumed their THC oil at least once per day, and therefore likely had relatively high tolerance levels. Another potential explanation is that experienced medicinal cannabis patients might put more effort into hazard perception to compensate for a perceived impairment when they know that they are under the influence of THC. If participants perceived that the test felt more effortful in the post-consumption condition, then this may explain why they rated their performance less favourably despite the lack of a measurable decline. In either case, our results align with a growing body of evidence demonstrating that chronic THC use can mitigate the acute effects of the substance on driving performance (Bosker et al. 2012; Brooks-Russell et al. 2021) and driving-related cognitive functions (Desrosiers et al. 2015; Ramaekers et al. 2011; Schwope et al. 2012). This highlights the need to also examine the effects of orally ingested THC on hazard perception among occasional or new users, such as medicinal cannabis patients in the early stages of their treatment, who may be yet to develop tolerance and/or compensatory strategies.

Dosage and symptom relief might have also influenced the present findings. First, the present study took a naturalistic approach towards dosing, with each participant consuming a single dose of their prescribed THC oil product. This led to an average of 10.80 mg THC (0.12 mg/kg) being consumed by the sample, which is approximately half of their self-reported typical single session dose (*M* = 19.57 mg, *SD* = 24.56) and far less than the total dose that participants reported typically consuming over course of a day via oil and/or flower products (*M* = 179.81 mg, *SD* = 178.13). Furthermore, this observed dose is within Australia’s therapeutic dosage range (5-20 mg; Arnold et al. 2020) and less than fixed flower doses typically investigated within the literature (e.g., Arkell et al. 2019; Ramaekers et al. 2006; Spindle et al. 2020). Experimental research administering similar THC doses have found either modest or no significant performance deficits on measures of cognitive functions that correlate with hazard perception skill (Gray et al. 2008; Schlienz et al. 2020; Spindle et al. 2021). These prior findings suggest that the average THC dose observed in this study may not have been sufficient to produce deficits in hazard perception performance following ingestion. Notably, a small subset of participants self-reported extremely high typical THC doses, with daily THC consumption (via both oil and flower products) estimates reaching up to 625 mg THC (albeit this could not be verified). Further research is needed to understand the impacts of regular THC ingestion among medicinal patients, who require their medication on a frequent and ongoing basis.

Second, participants in the present sample were also being treated for a range of conditions (including chronic pain, mental health disorders, and insomnia) that can independently have negative effects on cognitive function and driving capacity (Bulmash et al. 2006; Iezzi et al. 2004; Perrier et al. 2014; Sheline et al. 2006; Veldhuijzen et al. 2006). While research in clinical populations is limited, one study found that 20 mg of inhaled THC resulted in no deficits (or even modest improvements) in performance on measures of processing speed, attention, and executive functioning amongst a similarly mixed patient sample (Olla et al. 2021). It is therefore possible that our clinical sample experienced the alleviation of symptoms following their medication use, which in turn mitigated the extent to which THC affected performance. Nonetheless, further research that compares medicinal cannabis patients’ objective performance to general population norms is needed to elucidate the role of symptom relief in clinical populations that use cannabis for medicinal purposes.

An alternative explanation for the present findings is that the hazard perception test utilised in present study is not sufficiently sensitive to orally ingested THC. However, prior research has found video-based tests of hazard perception ability to be sensitive to other impairing substances (i.e., alcohol, West et al. 1993), as well as conditions such as acute mild traumatic brain injury (Preece et al. 2010), age-related decline in cognitive ability (Horswill et al. 2009), fatigue (Smith et al. 2009), distraction (Horswill and McKenna 1999), driver experience (Hill et al. 2019), and crash involvement (Horswill et al. 2015). For example, West et al. (1993) found that the consumption of moderate doses of alcohol between 0.04 and 0.06% delayed hazard perception response latencies by 1.1 s (West et al. 1993). That is, hazard perception tests have been found to be sensitive to a range of crash-related conditions. The present study is the first to examine the effects of acute oral THC consumption on hazard perception test performance, and hence we cannot rule out the possibility that higher doses of THC might significantly impair performance. This possibility could be examined in future research investigating the effects of a range of higher THC doses on hazard perception test scores. Furthermore, research comparing performance on video-based hazard perception tests with other cognitive and driving performance measures that have proven sensitive to acute THC administration (e.g., SDLP), is required.

It is also important to consider that driving is a complex task requiring a dynamic interplay of motor, visual, and cognitive functions (Anstey et al. 2005), and a hazard perception test cannot incorporate all the demands inherent to real-world driving. Nevertheless, scores from hazard perception tests developed using the same methodology as the test employed in the present study provide an effective means of measuring a driver’s “situation awareness on the road” (Horswill and McKenna 2004), and are supported by evidence of experience-related performance differences, associations with crash rates (both retrospectively and prospectively), and measures of real-world driving performance such as heavy-braking frequency (Hill et al. 2019; Horswill et al. 2015; Wetton et al. 2011).

We found speeding propensity to decrease following acute THC ingestion. This is in accord with prior findings that recreational cannabis users adjust longitudinal control behaviours as a way of compensating for potential deficits in driving capacity (Brands et al. 2019; Brooks-Russell et al. 2021; Hartman et al. 2015; Lenné et al. 2010). In a recent experimental study, Brooks-Russell et al. (2021) observed a decrease in driving speed in a simulator amongst daily, but not occasional, recreational cannabis users following inhalation of cannabis flower containing THC. As our sample primarily consumed their medication at least once a day, our results align with this finding and support the notion that cannabis usage patterns can influence engagement in certain compensatory behaviours.

Similarly, participants’ minimum comfortable following distance increased following acute oral THC consumption. Whilst this result is consistent with previous findings of increased following distance after THC administration in occasional recreational cannabis users (Hartman et al. 2015; Lenné et al. 2010), it contrasts with recent survey research in which more than half of a sample of medicinal cannabis patients denied leaving a larger gap between them and the vehicle in front of them (Arkell et al. 2020a). However, Horswill et al. (2020) note that one of the reasons for developing the test used in the present study was because text-based self-report questions have been found to be ineffective for assessing following distance behaviour, likely because it is difficult to make such judgements reliably without the visual context that the traffic clips provide. Further, in the present study, participants made these contextualised judgements both with and without having consumed THC, so that the difference in their responses could be compared directly.

Nevertheless, despite an increased crash risk for individuals with poor gap acceptance behaviour (McDowell et al. 1983; Tupper et al. 2011), there was no significant change in gap acceptance wait time. Taken together, our findings suggest that, although medicinal cannabis users may engage in compensatory strategies when they know that they are potentially under the influence of THC oil, they may be more inclined to compensate for cannabis impairment through alterations in speed and following distance (which are closely related driving behaviours), rather than gap acceptance.

Finally, data from the self-report items indicated that participants had little or no insight into the effects of THC on their driving skills and safety. Not only did participants as a group perceive a decline in their hazard perception skill performance that was not borne out by the objective data, but there was also no significant relationship between their self-rated and objective performance at either timepoint (baseline or the post-consumption). In addition, self-ratings of on-road driving skills and safety did not significantly change from baseline to post-consumption, after correcting for multiple tests. Without objective on-road driving data, it is difficult to determine the true accuracy of such ratings. Nevertheless, they are consistent with a lack of substantial change in objective hazard perception skill performance. However, it is worth noting that self-ratings for *predicting traffic conflicts* and *driving safety* were significantly lower in the post-consumption condition prior to correction for multiple comparisons. Interestingly, if the uncorrected finding for predicting traffic conflicts is regarded as potentially meaningful (e.g., as suggesting that the inclusion of this measure in future studies with larger samples or fewer measures may be warranted), it is consistent with participants’ perceptions of a decline in their hazard perception test performance. Prior survey research suggests that medicinal cannabis patients are confident in their ability to accurately recognise their own level of impairment (Arkell et al. 2020a), yet recent preliminary findings indicate that cannabis users may in fact have a limited capacity to recognise their own driving impairment while acutely affected by THC (Arkell et al. 2019; Marcotte et al. 2022). Building on this research, the present study is the first to reveal a poor correspondence between objective and perceived driving performance through a direct correlational analysis, thus providing further evidence that medicinal cannabis patients’ appraisals of driving safety are inaccurate, which may increase the risk of DUIC. Despite this, our sample exhibited a tendency toward risk-aversion, likely as a means of compensating for perceived effects on performance.

Importantly, the present sample’s inability to accurately appraise their hazard perception skill performance at either time point implicates generally poor self-monitoring skills rather than an acute disruption of judgement and awareness due to THC consumption. This is consistent with prior findings that the general population tends to have inflated self-ratings of driving ability that poorly correspond with objective measures (Freund et al. 2005; Horswill et al. 2004, 2013). Most of the sample also rated their hazard perception skill performance, and on-road driving skills and safety, as better than average in both conditions, which further reflects the self-enhancement bias that is common among all driver groups. Nonetheless, these findings do not exclude the possibility that judgement and awareness may also be affected at higher THC doses and in less tolerant individuals. Given that perceptions of safety have been found to influence driving under the influence of cannabis (Borodovsky et al. 2020; Jones et al. 2007; Malhotra et al. 2017; McDonald et al. 2021), the impact of prior beliefs and attitudes on objective performance and self-ratings could be investigated in future research.

This study has several important limitations that must be noted. First, although all participants reported complying with the requirement to abstain from cannabis products for 11.5 h, almost half tested positive to THC in oral fluid prior to each testing session. This indicates that some amount of residual THC was present in their systems, which may have had an effect on their neurocognitive state. However, it is unknown whether this use was recent, given that THC is a highly lipophilic compound that can remain above oral fluid detection thresholds for up to 3 days in heavy cannabis users (Niedbala et al. 2001; Odell et al. 2015). Furthermore, the absence of blood measures prevented us from quantifying THC concentrations and its relevant metabolites under each condition, as well as from examining their association with task performance. Second, there was a substantial amount of variability in the THC dose that each participant consumed. These products also differed in cannabis strain, which can influence THC-induced subjective effects and potentially task performance (Sholler et al. 2022). Although varying amounts of cannabidiol were also present in each product, this compound appears to have negligible effects on neurocognition and driving-related skills (Arkell et al. 2019; Englund et al. 2023; McCartney et al. 2022b). Third, post-consumption performance on the video-based assessments was only measured at one time point, making it difficult to determine if the peak effects of the THC were appropriately captured. Approximately twenty minutes of neuropsychological testing was also conducted prior to the commencement of these tasks. However, this may be less problematic for orally ingested THC, considering that its effects have been shown to last for up to 4 h (Schlienz et al. 2020; Spindle et al. 2021).

Finally, it is possible that performance on the video-based measures was affected by changes in clinical symptomatology (e.g., pain flare-ups), as various clinical conditions were present within the sample, and baseline and post-consumption evaluations occurred on separate days. Certainly, future research is needed to address these issues by utilising controlled THC/CBD doses and additional assessment time-points for objective and self-rated performance. Furthermore, it is recommended that future medicinal cannabis research measures clinical symptoms to control for their influence on performance outcomes, and possibly explores the role of symptom relief in mitigating the psychoactive effects of THC. Despite these limitations, the present study offers important preliminary insights into the effects of orally ingested THC oil on drivers’ hazard perception skill performance, risk-taking behaviours, and self-perceptions of driving skills and safety within medicinal cannabis populations.

## Conclusion

The present study used a within-subjects design to examine the acute effects of orally-ingested THC on hazard perception skill performance, driving-related risk-taking behaviours, and self-perceived driving skills and safety amongst a sample of medicinal cannabis patients. Whilst THC did not acutely affect hazard perception skill performance or gap acceptance in our sample, we found preliminary evidence of behavioural compensation strategies in medicinal cannabis patients through a decrease in speeding propensity and an increase in following distance. No relationship was found between self-rated and objective hazard perception performance, highlighting that the sample were not accurate in their appraisals of performance, irrespective of cannabis consumption. Future research is needed to further investigate the effects of THC on these driving-related skills and behaviours using a wider range of doses and administration methods, with populations of varying tolerance levels. As the uptake of medicinal cannabis increases throughout Australia and other jurisdictions globally, developing a comprehensive understanding of the acute effects of THC on both objective and perceived driving performance will be crucial for guiding future road safety legislation in this area.

## Data Availability

The authors do not have approval to share the data.
